# Supplementary material for: Evolutionary game analysis of supply chain financing for film and television enterprises considering co-production
Source: PLoS One. 2025 Dec 2;20(12):e0336232. doi: 10.1371/journal.pone.0336232 (PMC12671796; doi:10.1371/journal.pone.0336232)
Supplement: S1 File — (DOCX) [file pone.0336232.s001.docx]

**Code File for the Experimental Section**

You should install matlab2019a, and any other version may have errors.Open one of the .m files (the solving files and the main files) in Matlab2019a.

Press “Debug”-“run” and you can get the results.If there is a reminder, choose "Change MATLAB current directory".Go to the “Command Window”, input “clear all” and press Enter, then you can run the next .m file.

**(1)Run Figure2.m**

p=400,c=150,F=10,a=0.3,r=0.05,q=0.3,k=0.4

for g=230

[t,y]=ode45(@(t,y)sj1(t,y,a,p,c,F,r,q,g,k),[0 10],[0.5 0.5 0.5]);

plot3(y(:,1),y(:,2),y(:,3),'rh-');

hold on

end

for g=270

[t,y]=ode45(@(t,y)sj1(t,y,a,p,c,F,r,q,g,k),[0 10],[0.5 0.5 0.5]);

plot3(y(:,1),y(:,2),y(:,3),'gs-');

hold on

end

for g=350

[t,y]=ode45(@(t,y)sj1(t,y,a,p,c,F,r,q,g,k),[0 10],[0.5 0.5 0.5]);

plot3(y(:,1),y(:,2), y(:,3),'bo-');

hold on

end

axis([0 1 0 1 0 1])

set(gca,'XTick',[0:0.2:1],'YTick',[0:0.2:1] ,'ZTick',[0:0.2:1])

xlabel('$x$', 'interpreter','latex');

ylabel('$y$','interpreter','latex');

zlabel('$z$','interpreter','latex','rotation',360);

grid on

legend('g=230','g=270','g=350 ');

hold on

Then you can get Figure 2.

**(2)Run Figure3.m**

p=400,c=150,F=10,a=0.3,r=0.05,q=0.3,k=0.4

for g=100

[t,y]=ode45(@(t,y)sj1(t,y,a,p,c,F,r,q,g,k),[0 10],[0.5 0.5 0.5]);

plot3(y(:,1),y(:,2),y(:,3),'rh-');

hold on

end

for g=300

[t,y]=ode45(@(t,y)sj1(t,y,a,p,c,F,r,q,g,k),[0 10],[0.5 0.5 0.5]);

plot3(y(:,1),y(:,2),y(:,3),'gs-');

hold on

end

axis([0 1 0 1 0 1])

set(gca,'XTick',[0:0.2:1],'YTick',[0:0.2:1] ,'ZTick',[0:0.2:1])

xlabel('$x$', 'interpreter','latex');

ylabel('$y$','interpreter','latex');

zlabel('$z$','interpreter','latex','rotation',360);

grid on

legend('g=100','g=300');

hold on

Then you can get Figure 3.

**(3)Run Figure4.m**

p1=0.3,p2=0.3,p3=0.3;

a=0.3,p=400,c=150,F=10,r=0.05,q=0.3,g=250,k=0.4

[t,y]=ode45(@(t,y)sj1(t,y,a,p,c,F,r,q,g,k),[0 1],[p1 p2 p3]);

y1=y(:,1);

plot(t,y1,'--r');

hold on;

y2=y(:,2);

plot(t,y2,'*-k');

hold on;

y3=y(:,3);

plot(t,y3,'--g');

xlabel('t');

ylabel('p');

hold on;

legend('film and television enterprise','Sales Platform','The bank')

Note: p1, p2, p3 stands for X, Y, and Z

Then you can get Figure 4.

**(4)Run Figure5.m**

p=400,c=150,F=10,a=0.3,r=0.05,q=0.3,k=0.4

for g=100

[t,y]=ode45(@(t,y)sj1(t,y,a,p,c,F,r,q,g,k),[0 10],[0.5 0.5 0.5]);

plot3(y(:,1),y(:,2),y(:,3),'rh-');

hold on

end

for g=300

[t,y]=ode45(@(t,y)sj1(t,y,a,p,c,F,r,q,g,k),[0 10],[0.5 0.5 0.5]);

plot3(y(:,1),y(:,2),y(:,3),'gs-');

hold on

end

axis([0 1 0 1 0 1])

set(gca,'XTick',[0:0.2:1],'YTick',[0:0.2:1] ,'ZTick',[0:0.2:1])

xlabel('$x$', 'interpreter','latex');

ylabel('$y$','interpreter','latex');

zlabel('$z$','interpreter','latex','rotation',360);

grid on

legend('g=100','g=300');

hold on

Then you can get Figure 5.

**(5)Run Figure6.m**

p=400,c=150,g=300,a=0.3,r=0.05,q=0.3,k=0.4

for F=10

[t,y]=ode45(@(t,y)sj1(t,y,a,p,c,F,r,q,g,k),[0 10],[0.5 0.5 0.5]);

plot3(y(:,1),y(:,2),y(:,3),'rh-');

hold on

end

for F=80

[t,y]=ode45(@(t,y)sj1(t,y,a,p,c,F,r,q,g,k),[0 10],[0.5 0.5 0.5]);

plot3(y(:,1),y(:,2),y(:,3),'bo-');

hold on

end

for F=200

[t,y]=ode45(@(t,y)sj1(t,y,a,p,c,F,r,q,g,k),[0 10],[0.5 0.5 0.5]);

plot3(y(:,1),y(:,2),y(:,3),'gs-');

hold on

end

axis([0 1 0 1 0 1])

set(gca,'XTick',[0:0.2:1],'YTick',[0:0.2:1] ,'ZTick',[0:0.2:1])

xlabel('$x$', 'interpreter','latex');

ylabel('$y$','interpreter','latex');

zlabel('$z$','interpreter','latex','rotation',360);

grid on

legend('F=10','F=80','F=200');

hold on

Then you can get Figure 6.
