# Supplementary material for: Evolutionary game analysis of supply chain financing for film and television enterprises considering co-production
Source: PLoS One. 2025 Dec 2;20(12):e0336232. doi: 10.1371/journal.pone.0336232 (PMC12671796; doi:10.1371/journal.pone.0336232)
Supplement: Attachment — Submitted filename: comments.docx [file pone.0336232.s002.docx]

Referee report on “EVOLUTIONARY GAME ANALYSIS OF SUPPLY CHAIN FINANCING FOR FILM AND TELEVISION ENTERPRISES CONSIDERING CO-PRODUCTION”

Summary

Currently, many film and television enterprises struggle with insufficient funds during the visualization process. To address this issue, companies can opt to enter into coproduction agreements with sales platforms, implement supply chain financing, obtain financing through banks, or collaborate with all three parties simultaneously. The paper presents a tripartite evolutionary game model involving film and television enterprises, sales platforms, and banks and discusses the strategic choices of operation and the investment and financing behaviors of the three parties. Additionally, it analyzes film and television enterprises' capital, film production costs, copyright acquisition costs, and other factors and examines the impact of these factors on their strategic choices. The research provides theoretical support for further alleviating the financing difficulties of film and television enterprises and at the same time provides a theoretical basis for the cooperation among film and television enterprises, sales platforms, and banks. This topic is interesting and the work is carefully designed. However, there are still concerns as follows.

Major concerns:

1. In the introduction section, it seems more discussion is expected to explain the contribution of this work and the novel findings.
2. In section 2, there shall be the literature review which contains discussions about film and television enterprises.
3. In section 3, there is no discussion of the existing literature on model assumptions and the reasons why this article focuses on this design.

Minor concerns:

1. The formatting of the conclusion is a bit strange, which requires revision.
