# Supplementary material for: Evolutionary game analysis of supply chain financing for film and television enterprises considering co-production
Source: PLoS One. 2025 Dec 2;20(12):e0336232. doi: 10.1371/journal.pone.0336232 (PMC12671796; doi:10.1371/journal.pone.0336232)
Supplement: Attachment — Submitted filename: Response to Reviewers20241116.docx [file pone.0336232.s004.docx]

**Response to Reviewers**

We acknowledge the reviewers’ comments which are valuable in improving the quality of our manuscript. We have revised our manuscript addressing these comments or suggestions. The changes in the revised version are colored blue.

**Reviewer 1:**

The write up is excellent. The stage chosen is for film but can be easily applied to banking financing of other sectors. Paper is well written I have nothing more to add other than it would be good if the model is tested on actual data.

**Response:** Thank you very much for the insightful comments regarding the model validation. We fully agree that using real data to test the model can further enhance the empirical foundation of the research. Thus, in the section of “4. Simulation analysis”, we have added the actual data to explain the rationality of parameter settings as follows:

“Based on the data of Huayi Brothers Research Institute (2017), for a Hollywood film with proceed of $100 million, the average production cost is $30,482,379 and the average miscellaneous cost is $14,828,500, which means the operations cost as a percentage of revenue are about 40%; the average copyright acquisition cost is $51,642,345, which means the copyright cost as a percentage of revenue are about 50%. Based on the data of Weisaidi (2021), a classic Hollywood film’s proceed is about $400 million. Thus, we set $p=400,$ and the copyright acquisition cost is $g=200$, which is 50% of the proceed, and the Costs incurred by film and television enterprises for making the film is $c=150$, which is 37.5% of the proceed. The bank loan interest rate is assumed to be $r=0.05$, which is in line with reality.”

**Reviewer 2:**

The paper presents a tripartite evolutionary game model involving film and television enterprises, sales platforms, and banks and discusses the strategic choices of operation and the investment and financing behaviors of the three parties.It has a good structure and follows a scientific approach. The results are well interpreted, however, they lack discussion with other findings.

**Response:** Thank you very much for the valuable suggestion. We fully agree with your perspective that the discussion of our current results in relation to other relevant findings could be further strengthened. In the revised manuscript, the introduction section has been rewritten to re-examine the contributions and novel discoveries of the article. The literature review section has been expanded to include an overview of other studies that are relevant to or comparable with our research findings, as well as a discussion on the advantages of the research methodology employed in this paper.

**Reviewer 3:** Conclusively, my suggestion for this article is to accept it after a minor revision. This article has no methodological errors. However, I suggest the author provides the following revisions:

1. The literature review of this manuscript did not show the advantage of applying an evolutionary model to the studied problem. I suggest the author present this advantage for illustrating why an evolutionary model was employed in this article.

**Response:** Thank you very much for the valuable suggestion. In the revised version of this paper, the literature review section has been updated to articulate the distinctive advantages of introducing evolutionary game theory models into the research of film and television industry financing. The evolutionary model not only transcends the static constraints of traditional financing analysis frameworks but also delineates the evolutionary mechanisms of financing strategies employed by film and television enterprises. This approach offers a novel perspective and strategic recommendations for addressing the prevalent issues of difficult and expensive financing in the film and television industry.

2. I further recommend the author reviews the theoretical background of an evolutionary model. Just applying an evolutionary model is insufficient.

**Response:** Thank you very much for the valuable suggestion. In the introduction section, the valuable feedback and insights provided by the reviewer experts have been incorporated, leading to a detailed elaboration of the theoretical background of evolutionary game theory.

3. How to plot Figure 1 on the 10th page of this manuscript was not sufficiently explained. Please add the explanation.

**Response:** Thank you very much for pointing out the insufficient explanation of Figure 1 in the paper. In the revised manuscript, I have added a textual description of Figure 1, elaborating on its construction and its relation to the research conclusions, thereby enhancing its clarity and readability.

4. Are baseline models available for this article? I suggest the author may provide some baseline models.

**Response:** Thank you very much for the suggestion. This article studies the financing issues of film and television companies through an evolutionary game model, a method typically aimed at dynamic environments and strategy evolution processes. In contrast, benchmark models are generally used in relatively stable environments, making direct comparisons with static benchmark models challenging. Therefore, the article does not design a benchmark model.

**Reviewer 4:**

Referee report on “EVOLUTIONARY GAME ANALYSIS OF SUPPLY CHAIN FINANCING FOR FILM AND TELEVISION ENTERPRISES CONSIDERING CO-PRODUCTION”

Summary

Currently, many film and television enterprises struggle with insufficient funds during the visualization process. To address this issue, companies can opt to enter into coproduction agreements with sales platforms, implement supply chain financing, obtain financing through banks, or collaborate with all three parties simultaneously. The paper presents a tripartite evolutionary game model involving film and television enterprises, sales platforms, and banks and discusses the strategic choices of operation and the investment and financing behaviors of the three parties. Additionally, it analyzes film and television enterprises' capital, film production costs, copyright acquisition costs, and other factors and examines the impact of these factors on their strategic choices. The research provides theoretical support for further alleviating the financing difficulties of film and television enterprises and at the same time provides a theoretical basis for the cooperation among film and television enterprises, sales platforms, and banks. This topic is interesting and the work is carefully designed. However, there are still concerns as follows.

Major concerns:

1. In the introduction section, it seems more discussion is expected to explain the contribution of this work and the novel findings.

**Response:** Thank you very much for the valuable suggestion. In the revised manuscript, the introduction section has undergone a major revision. To better lead into the contributions and novel discoveries of this paper, the introduction now provides a thorough analysis of the theoretical background of evolutionary game theory. It explicitly outlines the unique contributions of this paper compared to existing studies, including the issues addressed, as well as the new perspectives and methodologies offered.

2. In section 2, there shall be the literature review which contains discussions about film and television enterprises.

**Response:** Thank you very much for the valuable suggestion. In the revised version, the second section has been adjusted to serve as a comprehensive literature review, which now includes additional relevant literature pertaining to film and television enterprises.

3. In section 3, there is no discussion of the existing literature on model assumptions and the reasons why this article focuses on this design.

**Response:** Thank you very much for the valuable suggestion. In Section “2.1 Model assumptions” of the revised version, we have added explanations to illustrate the rationality of model assumptions and have cited relevant references to support it.

Minor concerns:

1. The formatting of the conclusion is a bit strange, which requires revision.

**Response:** I'm very sorry for the inconvenience. I have rearranged and revised the conclusion part according to the requirements of the journal format.
