# Supplementary material for: Evolutionary game analysis of supply chain financing for film and television enterprises considering co-production
Source: PLoS One. 2025 Dec 2;20(12):e0336232. doi: 10.1371/journal.pone.0336232 (PMC12671796; doi:10.1371/journal.pone.0336232)
Supplement: Attachment — Submitted filename: Response to Reviewers.docx [file pone.0336232.s005.docx]

**Response to Reviewers**

We acknowledge the reviewers’ comments which are valuable in improving the quality of our manuscript. We have revised our manuscript addressing these comments or suggestions. The changes in the revised version are colored blue.

**Reviewer 1:**

Now the paper is excellent. Nifty mathematics but in real life things work when there is common interest between all parties. Don’t need mathematics to tell us that.

**Response:** Thank you very much for your recognition of our paper. Indeed, in real life, things work when there is common interest between all parties, which is consistent with our game equilibrium. However, the overall interests of the film and television enterprise, the sales platform and the bank under the game equilibrium are not optimal. How to design the financing strategies to achieve a win-win situation for all three parties requires mathematical analysis methods.

**Reviewer 2:**

It is evident that the authors have meticulously revised and refined the reviewers’ comments, thereby enhancing the quality of the revised manuscript. In my opinion, this renders it highly suitable for immediate publication.

**Response:** Thank you very much for your valuable suggestions mentioned earlier. Thank you very much for the acceptance of our paper.

**Reviewer 3:**

Conclusively, my suggestion for this article is to accept it after a minor revision. I suggest the authors provide the following changes:
1. In the literature review of this manuscript, I have read many published papers, which considered the main problem considered in this article.
Nevertheless, these previous papers seem not to be devoted to the game theory. Why did the authors choose the game theory to implement the current study?

**Response:** Thank you very much for the valuable suggestion. In the revised version of this paper, we have added an overall review of the existing literature and an explanation of why game theory was applied in our study. The specific description is as follows:

“*However, most of the existing literature uses qualitative methods such as policy and case studies to analyze the factors influencing the financing of film and television enterprises and the countermeasures to alleviate the problem of financing difficulties for small and medium-sized enterprises; there are also studies using empirical research methods to analyze the factors influencing the success of crowdfunding for film and television enterprises and the paths for film and television enterprises to implement crowdfunding financing. The existing research methods are limited to the decision-making of individual enterprises and cannot study the strategic interactions among supply chain member enterprises and banks. Therefore, this paper uses the game theory to study the cooperative behavior between film and television enterprises and the platform, as well as the lending behavior of banks. This can break through the limitations of the one-way causal relationship in traditional decision-making theories and provide a quantifiable framework for the dynamic evolution of complex systems.*”

2. The contribution of this article is unclear. It looks like that the authors just wanted to develop a new application of the game theory. The authors can define the contribution in the end of the Introduction section.
After complementing the above two questions, the review can end.

**Response:** Thank you very much for the valuable suggestion. In the revised version of this paper, we have added the contribution in the end of the Introduction section. The specific description is as follows:

“*The contribution of this paper lies in revealing the influence mechanism of the cooperation between film and television enterprises and sales platforms on the lending behavior of banks, as well as the effects of factors such as negotiation ability, the cost of copyright acquisition and the proportion of equity in joint production on dynamic evolution, providing suggestions for the selection of the optimal cooperation model for film and television enterprises.*”
