# Supplementary material for: Evolutionary game analysis of supply chain financing for film and television enterprises considering co-production
Source: PLoS One. 2025 Dec 2;20(12):e0336232. doi: 10.1371/journal.pone.0336232 (PMC12671796; doi:10.1371/journal.pone.0336232)
Supplement: Attachment — Submitted filename: Response_to_Reviewers_auresp_3.docx [file pone.0336232.s006.docx]

**Response to Reviewers**

We acknowledge the reviewers’ comments which are valuable in improving the quality of our manuscript. We have revised our manuscript addressing these comments or suggestions. The changes in the revised version are colored blue.

**Reviewer 2:**

It is evident that the authors have refined and augmented the full paper in response to the feedback provided by the reviewers, and it is my conviction that the revised manuscript fulfils the criteria for publication.

**Response:** Thank you very much for your valuable suggestions mentioned earlier. Thank you very much for the acceptance of our paper.

**Reviewer 3:**

I have only one comment remaining. This comment is optional. The review can end after considering this opinion. The specialty of this article is the development of an evolutionary game model. As a reader of this article, I would like to understand the advantage of adopting an evolutionary game model in decision-making.

**Response:** Thank you very much for the valuable suggestion. In the revised version of this paper, we have added the explanations regarding the advantages of evolutionary game model in two places.

The first place is at the end of the “Introduction”:

“*We use the evolutionary game model to reveal the learning behaviors of irrational decision-makers and the dynamic characteristics of the equilibrium strategies.”*

The second place is at the end of the “Literature review”:

“*Furthermore, the main advantage of the evolutionary game model lies in its dynamic adaptability. It simulates the real process in which decision-makers adjust their strategies through learning.”*
